# Reflex Dental Pain

**Published:** 1899-10

**Authors:** S. F. Heverly

**Affiliations:** Cascade, Iowa.


ARTICLE VII.

### REFLEX DENTAL PAIN.

S. F. HEVERLY, D. D. S., CASCADE, IOWA.

Miss L. Teacher, aged about twenty-three, came to my office Saturday evening, June 10, complaining of severe pain in four upper teeth, viz., left lateral incisor, cuspid, and first and second bicuspids, which, she said, had appeared about two days previously, and had been so severe as to not only keep her awake at night, but to compel her to cry from the pain.

I immediately suspected initial stages of alveolar abscess, and sought the history of the fillings which she had in incisor and both bicuspids. She admitted that the incisor had been very sensitive when filled several years before; did not remember tnat any of them had had root fillings. All four felt longer than usual, and were so sore that she could not use them. On percussion I found that they responded to the test for pericementitis—but all were equally sensitive—the cuspid which was apparently sound, was as sore as any.

I was not satisfied that I knew the exact cause, so I painted the gums thoroughly with creosote and iodine, prescribed phenacetine and morphine sulphate in doses of five grains of the former to one-half grain of the latter to be taken every three hours until better, and sent her home with the request to call next morning. Sunday morning she appeared and reported no relief; had not been able to sleep; pain continued as before. I made a thorough examination, but could discover no particular change, but supposed the chief offender to be the incisor which had two gold fillings—mesial and distal—so I proceeded to open into the pulp chamber. But when I had passed the enamel, the tooth was so sensitive that I could go no further.

I stopped and directed my attention to a canker sore on the mucous membrane of the lip, just opposite the cuspid tooth, which was very angry looking and had caused the lip to swell sufficiently to be noticeable. I cauterized it thoroughly with nitric acid, full strength, sealed opening I had made in lateral; and sent her away with instructions to call next morning.

Monday morning she appeared and reported that the pain had continued all day and night until about two o'clock a. m., then had ceased and she had been free from pain ever since, but the bicuspids were still sore. Examination showed the swelling gone and sore much reduced. I gave it another treatment of H N O3, applied capsicum pad over region of roots of bicuspids and dismissed patient with instructions to call after school, if the pain continued. She did not return, and I concluded that the canker sore was the cause of all the trouble; that the pain in teeth was reflex pain, and that the nitric acid had produced a cure.—*Items of Interest.*

